# Neuropathological findings suggestive for a stroke in an alpaca (*Vicugna pacos*)

**DOI:** 10.1186/s13028-018-0438-9

**Published:** 2019-01-03

**Authors:** Sandra Schöniger, Enrika Schütze, Dominik Michalski, Joana Puchta, Matthias Kaiser, Wolfgang Härtig

**Affiliations:** 1 Institute of Veterinary Pathology, Faculty of Veterinary Medicine, Leipzig University, Leipzig, Germany; 2 Department of Neurology, Medical Faculty, Leipzig University, Leipzig, Germany; 3 Paul Flechsig Institute for Brain Research, Medical Faculty, Leipzig University, Leipzig, Germany; 4 Clinic for Ruminants and Swine, Faculty of Veterinary Medicine, Leipzig University, Leipzig, Germany

**Keywords:** Alpaca, Fluorescence labeling, Focal brain lesion, Ischemic infarction, Pathology, *Vicugna pacos*

## Abstract

**Background:**

This case report describes a focal brain lesion in an alpaca (*Vicugna pacos*). Although this is a restricted study based on a single animal, neuropathological features are reported that are most likely attributed to a vascular event with either ischemic or hemorrhagic pathology. Concerning translational issues, these findings extend neurovascular unit concept to the alpacas’ brain and qualify a larger panel of stroke tissue markers for further exploration of ischemic or hemorrhagic consequences beyond the usually used small animal models in stroke research.

**Case presentation:**

A brain lesion indicative of a stroke was diagnosed in a 3-year-old female alpaca as an incidental finding during a *post mortem* examination. The rostral portion of the right frontal lobe contained a 1.0 × 1.5 × 1.7 cm lesion that extended immediately to the overlying leptomeninges. Microscopically, it was composed of liquefactive necrosis with cholesterol crystal deposition and associated granulomatous inflammation as well as vascularized fibrous connective tissue rimmed by proliferated astrocytes. Multiple fluorescence labeling of the affected brain regions revealed strong microgliosis as shown by immunostaining of the ionized calcium binding adapter molecule 1 and astrogliosis as demonstrated by enhanced immunoreactivity for glial fibrillary acidic protein. In parallel, a drastic neuronal loss was detected by considerably diminished immunolabeling of neuronal nuclei. Concomitantly, up-regulated immunoreactivities for collagen IV and neurofilament light chains were found in the affected tissues, indicating vascular and cytoskeletal reactions.

**Conclusions:**

Driven by these neuropathological features, the incidental brain lesion found in this alpaca strongly suggests an ischemic or hemorrhagic etiology. However, since typical hallmarks became verifiable as previously described for other species affected by focal cerebral ischemia, the lesion is more likely related to an ischemic event. Nevertheless, as such cellular alterations might be difficult to distinguish from other brain lesions as for instance caused by inflammatory processes, adjuvant observations and species-related features need to be considered for etiological interpretations. Indeed, the lack of neurological deficits is likely attributed to the location of the lesion within the rostral aspect of the right frontal lobe of the alpacas’ brain. Further, fibroblast migration from the meninges likely caused the intralesional scar formation.

## Background

In human beings, ischemic stroke represents a major cause of permanent disability and death [1]. The etiology is often multifactorial with the contribution of predisposing conditions such as atherosclerosis, diabetes mellitus, arterial hypertension, hypercoagulability and cardiac arrhythmia [2].

With the intention to explore the underlying mechanisms for stroke, several animal models were developed during the last decades, while limitations still exist referring to the transferability to the human condition [3, 4]. As a possible reason, the brain anatomy of the used animal species has been discussed in a critical manner, leading to the recommendation to consider gyrencephalic species in more advanced study phases [5].

On the cellular level, experimental stroke was demonstrated to cause remarkable morphological changes in various neural cells and the extracellular matrix, supporting the concept of the neurovascular unit that describes simultaneous ischemic reactions in a functionally associated complex of diverse cell populations [6, 7].

Cardiovascular accidents in domestic animals are less commonly reported and most cases of spontaneous brain infarcts have been described for cats and dogs [8–10]. In dogs, they are most frequently attributed to different types of emboli, although a hypercoagulable status is also recognized as an important factor [11, 12]. In comparison, cardiomyopathy-induced thromboembolism and hypertension related to renal failure are the most frequent risk factors for brain infarcts in cats [13].

The aim of this study was to explore a focal brain lesion in an alpaca (*Vicugna pacos*) while providing a macroscopic and concise histochemical characterization of affected brain tissue with a special emphasize to the neurovascular unit concept and, thereby, some arising parallels with an ischemic infarction.

This is the first reported case of a stroke in an alpaca. Consequently, the investigation into the resulting alterations of cellular elements and the vasculature will extend the translational perspective of neurovascular changes due to focal brain injury such as ischemia or hemorrhage to the alpacas’ brain, and thus beyond the usually applied small animal models in the field of stroke research.

## Case presentation

A 3-year-old female alpaca was submitted to the Clinic for Ruminants and Swine, Leipzig University with a clinical history of markedly reduced food and water intake. At admission the animal presented dehydrated and showed salivation as well as inappetence. Initially, feces were dry with mucous and traces of blood; after 1–2 days defecation was watery and bloody and then nearly completely ceased. Since its admission to the clinic, the alpaca had received intravenous infusions of glucose and electrolyte solutions as well as antiphlogistic and antibiotic treatments. First the general condition of the alpaca was stable, and it showed intake of solely a little bit grass and some water. One day prior to its death, its general condition markedly deteriorated; the alpaca was recumbent with colicky abdominal pain. The animal deceased spontaneously 4 days after its admission to the clinic and was submitted for a complete *post mortem* examination.

**Fig. 1:**
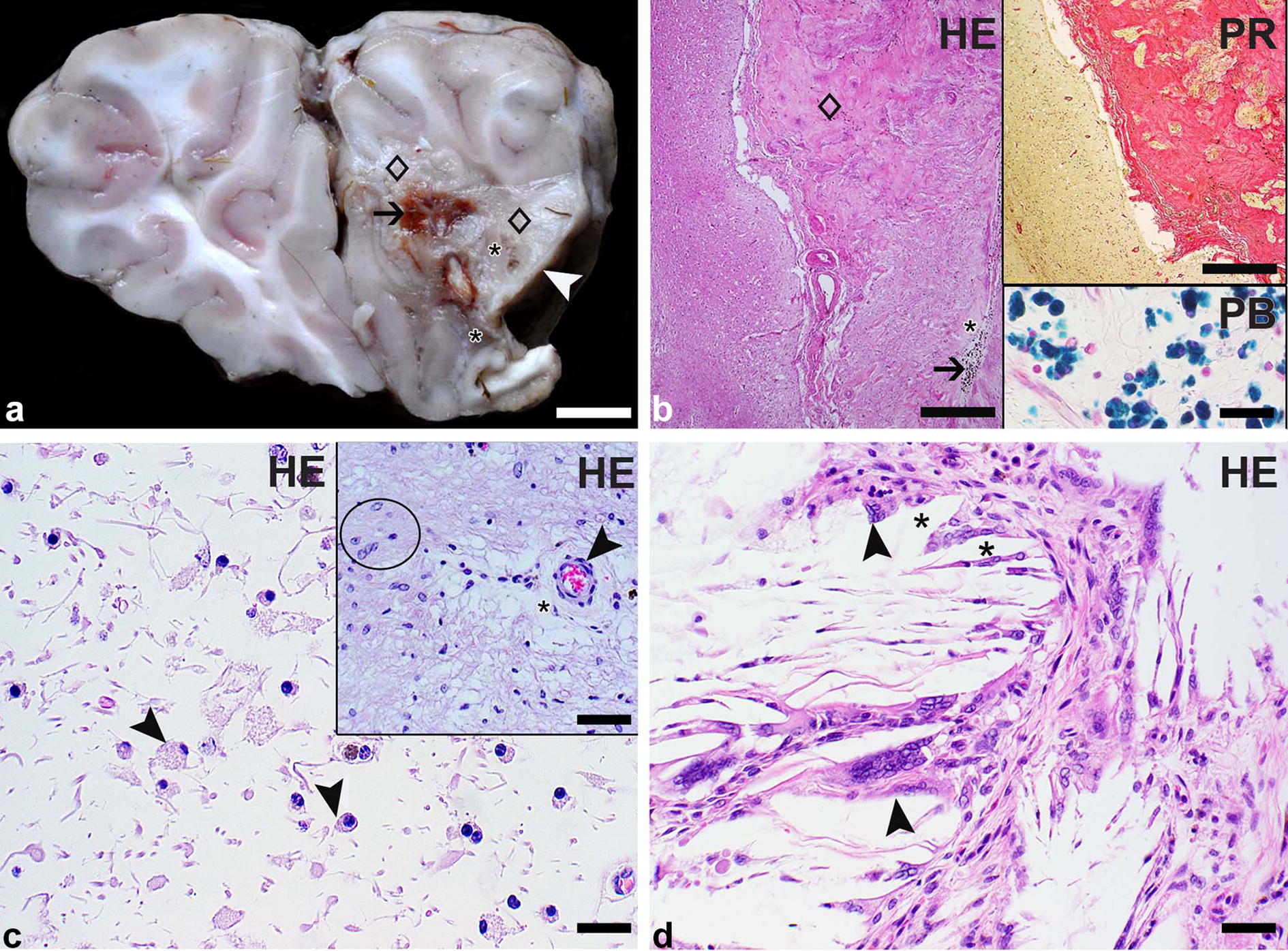
Focal brain lesion in an alpaca (*Vicugna pacos*): macroscopic and light microscopic findings. **a** Transverse section through the frontal lobes: The right frontal lobe contains a focal lesion that extends immediately to the leptomeninges of the lateral and medial sites (white arrowhead). The lesion is characterized by the loss of normal brain architecture within cortex and medulla. It consists of firm whitish-beige tissue (diamonds) as well as small areas of soft beige tissue (asterisks) and shows multifocal brownish discoloration (arrow). Bar: 1 cm. **b** By the microscopic examination, the firm whitish-beige tissue is identified as vascularized fibrous connective tissue (diamond) and the soft beige areas represent liquefactive necrosis (asterisk). The multifocal brown discoloration is attributed to the presence of sheets of siderocytes (arrow) revealed by hematoxylin–eosin (HE) staining. Bar: 200 µm. Upper inset: The fibrous connective tissue is highlighted in red color using a picrosirius red stain. Bar: 200 µm. Lower inset: The siderocytes are marked in blue color with a Prussian blue stain. Bar: 20 µm. **c** The area of liquefactive necrosis is infiltrated by moderate numbers of gitter cells (arrowheads) as shown by HE staining. Bar: 20 µm. Inset: Areas of necrosis and scar tissue formation are bordered by proliferated astrocytes (circle). An intralesional vessel (arrowhead) shows moderate perivascular edema (asterisk) visualized by HE staining. Bar: 40 µm. **d** The necrotic area contains aggregates of clear needle-like spaces consistent with cholesterol crystals (asterisks) surrounded by multinucleated histiocytic giant cells (arrowheads) as demonstrated by HE staining. Bar: 20 µm

At necropsy, the carcass was found in a good nutritional condition. The subcutis, the parietal and pulmonary pleura as well as the heart showed multiple endo- and epicardial petechial to ecchymotic hemorrhages. The myocardium, pulmonary artery, aorta, liver and kidneys showed no gross lesions. The abdominal cavity contained 40 mL of serosanguinous effusion. There was a moderate fibrinous to fibroblastic peritonitis with adhesions between the intestinal loops as well as well as the stomach. Multifocal moderate acute serosal hemorrhages were observed as well. The mucosa of the 2nd and 3rd stomach compartment (C2 and C3) was diffusely dark red indicative of acute gastritis. Jejunum, ileum and colon displayed a marked acute to subacute diphtheroid-necrotizing enteritis. Mesenteric lymph nodes were moderately enlarged. Lungs were incompletely collapsed with acute alveolar edema. The right frontal lobe contained a 1.5 × 1.0 × 1.7 cm lesion with loss of normal cortical and medullary architecture that extended within the meninges. In the center of the lesion, the brain tissue was beige and soft with multifocal mild cavitation and contained a few small foci of brownish discoloration. The peripheral parts of the lesion were composed of firm whitish-beige tissue interpreted as fibrous connective tissue (Fig. 1a).

Samples of multiple organs including the stomach compartments C2 and C3, small and large intestines, mesenteric lymph nodes, lungs and brain (cerebrum with the lesion, additional parts of the ipsi- and contralateral cerebrum, hippocampus, cerebellum, midbrain and medulla oblongata) were fixed in 10% neutral buffered formalin, processed routinely and embedded in paraffin for microscopic examination. Two µm-thick sections were cut with a HM 400R Manual Sliding Microtome (Microm, Heidelberg, Germany). The microscopic evaluation was performed on hematoxylin–eosin stained tissue sections. For a better characterization of the brain lesions, a picrosirius red staining [14] for the detection of fibrous connective tissue and a Prussian blue staining [15] to identify hemosiderin pigment were used.

## Histopathological findings

In C2 and C3, there was a mild diffuse acute fibrinous gastritis with intravascular fibrin thrombi. The marked acute diphtheroid-necrotizing enteritis was also associated with intravascular fibrin thrombi and a multifocal mild fibrinous serositis. Additionally, some intralesional vessels showed segmental to diffuse fibrinoid necrosis of their walls. Enlarged mesenteric lymph nodes had a diffuse marked acute suppurative lymphadenitis. Within the lung tissue, alveolar walls were lined by hyaline membranes and their lumens contained some fibrin stands and edema fluid.

In the right frontal lobe, the lesion was located immediately adjacent to the anterior horn of the right ventricle and extended into the lateral and medial leptomeninges. It was composed of immature and mature fibrous connective tissue with a moderate vascular proliferation and several areas of liquefactive necrosis; neurons were completely lost. The fibrous connective tissue was highlighted by a picrosirius red staining. Necrotic areas were infiltrated by moderate numbers of gitter cells as well as by a few gemistocytes and contained variably-sized cystic spaces. There were multifocal small aggregates of needle-like clear structures (consistent with cholesterol crystals) surrounded by a moderate granulomatous inflammation characterized by the presence of histiocytic cells including several multinucleate giant cells. The grossly noted brownish discoloration was attributed to the presence of sheets of mononuclear cells (microglia/macrophages) containing intracytoplasmic brown coarse pigment that was identified as hemosiderin by the Prussian blue staining. The lesion was rimmed by proliferated astrocytes (Fig. 1b–d). Intralesional vessels were characterized by surrounding empty spaces consistent with moderate perivascular edema. However, intralesional vessels and vessels of examined unaltered brain areas showed no evidence of intravascular thrombi, vasculitis or degenerative changes. A thorough microscopic evaluation of the entire brain area surrounding the lesion as well as representative locations of the remaining grossly unaltered brain revealed also no vascular changes; in particular, no vascular obstruction was detected. The immediately adjacent cortex contained some neurons with mild satellitosis.

To characterize in detail the cellular and vascular alterations, brain sections containing the lesion and adjacent brain parenchyma were examined by triple fluorescence staining. The following primary markers were applied (Table 1): ionized calcium binding adapter protein 1 (Iba) as a marker of microglia/macrophages, *Solanum tuberosum* lectin (STL) to visualize endothelial cells and microglia/macrophages, glial fibrillary acidic protein (GFAP) to stain astrocytic cell bodies, aquaporin 4 (AQP4) to detect astrocytic endfeet, collagen IV as a component of the vascular basement membrane, neuronal nuclear antigen (NeuN) to label neuronal nuclei and neurofilament light chain (NF-L) to verify the expression of this structural protein.

**Table 1:** Lectin-histochemical staining with biotinylated STL and Cy5-streptavidin combined with immunofluorescence double labelling of alpaca forebrain tissue sections

First primary antibodies	First visualising immunoreagents	Second primary antibodies	Second visualising immunoreagents
Rabbit-anti-Iba (1:400; Synaptic Systems, Göttingen, Germany)	Cy3-donkey-anti-rabbit IgG	Guinea pig-anti-GFAP (1:200; Synaptic Systems)	Cy2-donkey-anti-guinea pig IgG
Rabbit-anti-AQP4 (1:200; Alomone, Jerusalem, Israel)	Cy3-donkey-anti-rabbit IgG	Guinea pig-anti-GFAP (1:200; Synaptic Systems)	Cy2-donkey-anti-guinea pig IgG
Rabbit-anti-Iba (1:400; Synaptic Systems)	Cy3-donkey-antirabbit IgG	Guinea pig-anti-NeuN (1:200, Synaptic Systems)	Cy2-donkey-anti-guinea pig IgG
Rabbit-anti-NF-L (1:200; Synaptic Systems)	Cy3-donkey-anti-rabbit IgG	Goat-anti-collagen IV (1:50; Merck Millipore, Billerica, MA, USA)	Cy2-donkey-anti-goat IgG

Biotinylated STL was obtained from Vector Laboratories (Burlingame, CA, USA) and applied at 20 µg/mL for 20 h. All fluorescent immunoreagents were supplied by Dianova (Hamburg, Germany) and used at 20 µg/mL for 1 h

*STL Solanum tuberosum* agglutinin (= potato lectin), *Iba* ionized calcium binding adapter molecule-1, *GFAP* glial fibrillary acidic protein, *AQP4* aquaporin-4, *NeuN* neuronal nuclei, *NF-L* neurofilament, light chain

After deparaffinization, sections were heated for 25 min at 94 °C in 0.1 M citrate buffer, pH 6, for antigen retrieval. After three rinses with 0.1 M Tris-buffered saline (TBS), pH 7.4, tissue sections were incubated with different combinations of three primary markers (diluted in the blocking solution) for 20 h at room temperature (around 20 °C) as summarized in Table 1. Subsequently, tissue sections were washed with TBS and a mixture of three different fluorochromated secondary immunoreagents (Table 1) was applied for 1 h followed by extensive rinsing with TBS and two short washes with distilled water. Finally, sections were rinsed in distilled water, air dried and coverslipped with Entellan in toluene (Merck, Darmstadt, Germany). The omission of primary antibodies in control experiments resulted in the expected absence of any cellular labeling. The stained sections were screened with an Axioplan fluorescence microscope (Zeiss). All pictures were made with a Biorevo BZ-9000 microscope (Keyence, Neu-Isenburg, Germany).

**Fig. 2:**
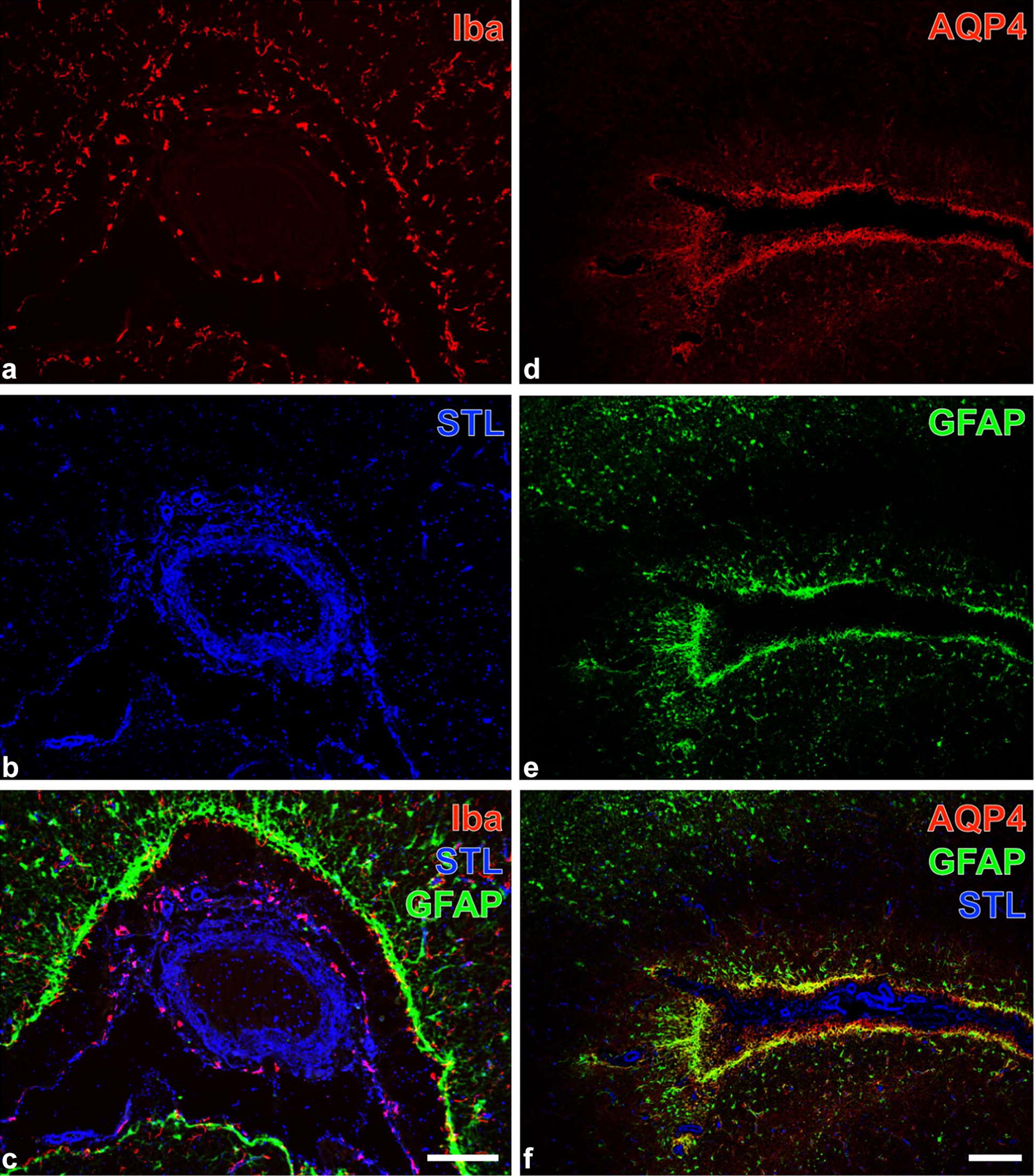
Focal brain lesion in an alpaca (*Vicugna pacos*): Fluorescence labeling to characterize intralesional microglia/macrophages and astroglia. **a**–**c** Triple fluorescence staining of microglia/macrophages (Iba/STL), vessels (STL) and astrocytes (GFAP). Red fluorescent Cy3-immunolabeling of Iba clearly demonstrates ameboid microglia/macrophages (**a**) surrounding a large vessel visualized by Cy5-marked binding sites for STL (**b**, color-coded in blue). The merged staining patterns (**c**) also display an enhanced GFAP-immunoreactivity (Cy2, green) at the vascular border. **d**–**f** Simultaneous staining of astroglial endfeet (AQP4) and astrocytic intermediate filaments (GFAP) combined with vessel detection (STL) in severely affected tissue. In parallel, the concomitantly enhanced immunoreactivities for AQP4 (**d**, Cy3) and GFAP (**e**, Cy2) surround a STL-stained large vessel (Cy5, color-coded in blue) as shown in (**f**). Bars: **c** (also valid for **a** and **b**): 100 µm, **f** (also valid for **d** and **e**): 150 µm

**Fig. 3:**
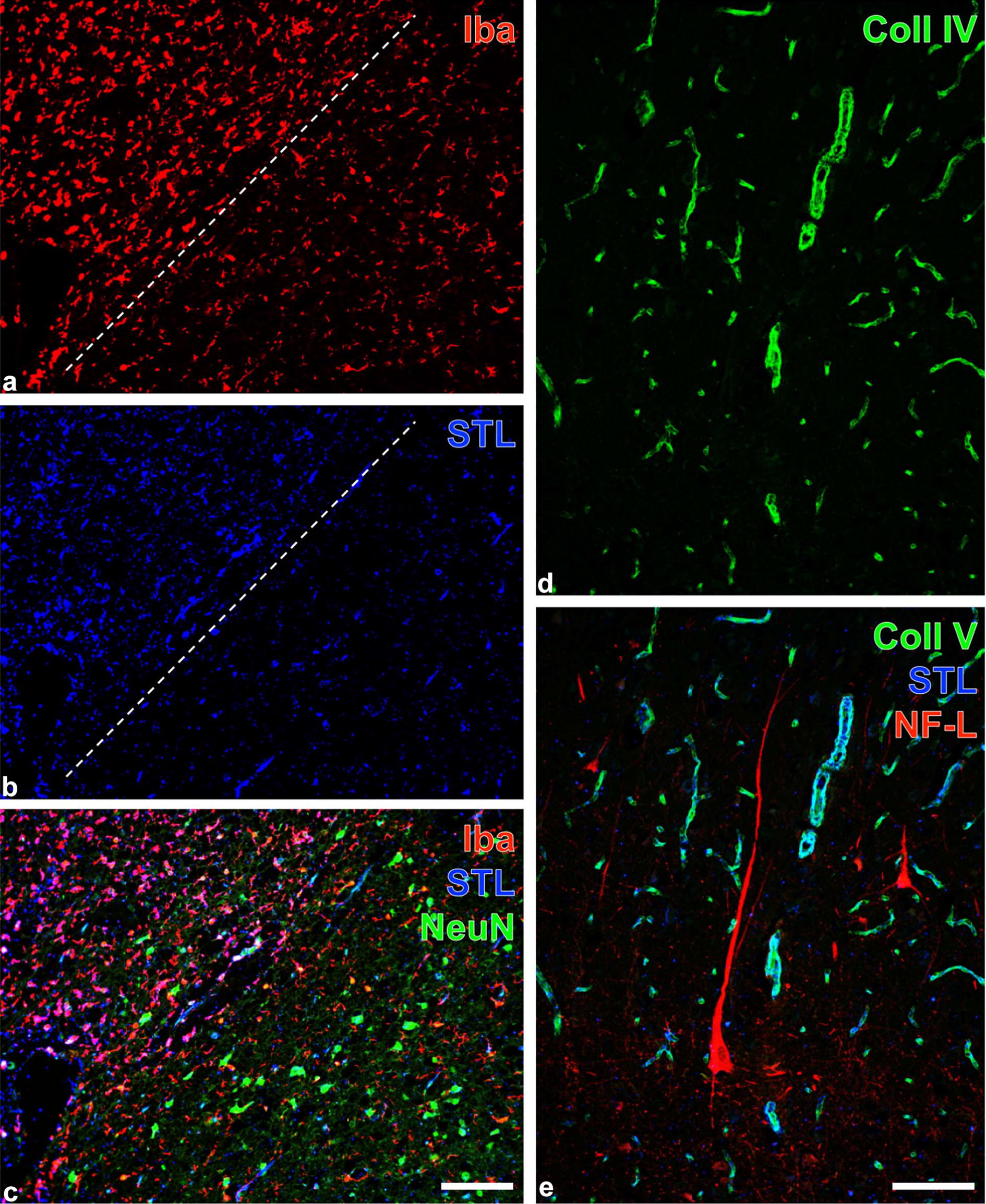
Focal brain lesion in an alpaca (*Vicugna pacos*): Fluorescence labeling to illustrate lesions at the border of the damaged tissue (**a**–**c**) as well as in in the core of affected neocortical brain regions (**d**, **e**). (**a**–**c**): Double staining of microglia/macrophages (Iba/STL) and vascular endothelial cells (STL) combined with the detection of neuronal nuclei (NeuN). The dashed line (**a**, **b**) separates the altered tissue (upper left part) from nonaffected brain parenchyma. In the upper left part (**a**), Iba-immunolabeling (Cy3, red fluorescence) reveals ameboid microglia/macrophages, simultaneously visualized in (**b**) by STL-binding sites (Cy5-staining, color-coded in blue). Additionally, the lectin-histochemical Cy5-staining (color-coded in blue) detects some endothelial cells. The merged staining patterns of Iba, STL and NeuN in (**c**) clearly show the neuronal loss in the gliotic tissue. (**d**, **e**): Simultaneous detection of the lesion markers collagen IV as component of the vascular basement membrane and neuronal NF-L counterstained by STL detecting vascular endothelial cells. In **d**, strong Cy2-immunostaining of collagen IV (Coll, Cy2, green) indicates lesioned tissue, whereas in (**e**) the merged staining patterns of Coll (Cy2, green) and STL (Cy5, color-coded in blue) results in the turquoise appearance of vessels. These are located in close vicinity to pyramidal cells displaying enhanced NF-L-immunoreactivity (Cy3, red). Scale bars: **c** (also valid for **a** and **b**): 100 µm, **d** (also valid for **e**): 150 µm

Four variants of triple fluorescence labeling contributed to the further histopathological characterization of the lesions. The affected tissues contained ameboid microglia/macrophages as revealed by Iba-immunolabeling (Fig. 2a) and lectin-histochemical STL-staining (Fig. 2b) primarily visualizing endothelial cells, as well as peripherally located, activated GFAP-immunopositive astroglia (Fig. 2c). Further, the lesioned tissue displayed strong AQP-4-immunolabeling of astroglial endfeet (Fig. 2d) allocated with GFAP-marked cell bodies (Fig. 2e) around a large STL-positive vessel (Fig. 2f). Additionally, regions exhibiting ameboid microglia/macrophages (Fig. 3a, b) were largely devoid of NeuN-immunopositive neurons as shown in Fig. 3c. Moreover, enhanced immunoreactivities for collagen IV and NF-L became visible in the affected tissue (Fig. 3d, e), further evidencing remarkable changes to the vascular and neuronal compartment of the neurovascular unit.

## Discussion and conclusions

The focal brain lesion that was incidentally found in this alpaca exhibited histopathological features including intralesional cellular and vascular changes that are strongly suggestive for a stroke with an either ischemic or hemorrhagic nature.

Generally, an ischemic infarct is typically caused by arterial occlusion with a thrombus or an embolus [11]. In the present case, a thrombotic event appears unlikely, since the vessel walls in the alpaca brain showed neither inflammatory nor degenerative changes. Instead, an embolus is regarded as the most likely cause for the observed lesion. Besides a blood clot, a septic (bacterial) or a parasitic embolus has to be considered as well [11]. *Elaeophora schneideri*, the arterial worm of mule deer and black-tailed deer, has been suspected as possible causative agents for brain infarctions in alpacas and llamas as well [16]. This parasite, however, is restricted to North America, and to our knowledge no confirmed cases of brain infarcts related to intravascular parasites have been reported in alpacas or llamas. The failure to detect a thromboembolic event at the time of the post mortem investigation may be explained by the chronicity of the lesion [10].

Nevertheless, a primary hemorrhagic stroke needs to be considered as the main differential diagnosis given the fact of absence of an embolus. It appears unlikely, however, that the brain lesion represents the chronic stage of a focal septic encephalitis due to the absence of neutrophilic infiltrates and/or abscessation [17]. In addition, it is unlikely attributed to parasite migration tracts, as these are usually characterized by foci of necrosis and/or fibrosis with associated mixed cellular inflammation including lymphocytes, plasma cells and eosinophils; intralesional parasites may be observed [18]. The lesion in the alpaca brain, however, was devoid of lymphocytes, plasma cells and eosinophils.

In contrast to the observed lesions, i.e. occasional cholesterol crystal deposits and associated granulomatous inflammation within a larger area of necrosis and fibrosis, xanthogranulomas are inflammatory tumor-like lesions consisting of foamy histiocytes (xanthoma cells), cholesterol crystals, multinucleated giant cells, siderocytes and fibrosis [19].

Notably, the described histopathological alterations were also observed in previous own studies focused on the neurovascular unit after experimental stroke in rodents, sheep and human autoptic tissue. In detail, applying two rat models of stroke, ischemia-induced micro- and astrogliosis with enhanced GFAP- and AQP4-staining was shown simultaneously to drastically diminished NeuN-immunopositive neurons in infarcted regions [20, 21]. Further, a remarkable increase of collagen IV-immunoreactivity as a histopathological feature of altered vessel integrity due to ischemia was described in filament-based stroke models applied to the mouse [22] and the rat [21]. Concomitantly enhanced immunolabeling of NF-L had been found after experimental stroke in rodents and sheep as well as in human stroke cases [23, 24], indicating a relevant affection of cytoskeletal elements due to the ischemic stimulus. Instead, such histological alterations strongly resembled ischemic stroke lesions in human beings [25] and different animal models [26, 27]. However, based on the presence of hemosiderin-laden microglia/macrophages, the hemorrhage nature of the found lesion needs to be discussed, but such cells were also observed in experimentally induced ischemic brain infarcts in sheep [26].

The term stroke refers to an ischemic or hemorrhagic brain injury in association with overt neurological deficits such as sensory and motor deficits and/or pain [10, 11, 25]. Notably, the presented alpaca did not show obvious neurological symptoms, which is likely attributed to the location of the brain lesion. In detail, if ischemia or hemorrhage occurs in brain regions that do not impact motoric or sensory functions, typical clinical symptoms are missing. For these cases, the designation silent stroke might be applied [28]. In human beings, injury to the prefrontal cortex can result in cognitive dysfunction and changes of personality traits [29, 30]. Accordingly, after experimental frontal lobotomy, dogs showed behavioral changes as well in particular an alleviation of aggressive behavior towards other dogs [31].

In the present case, the brain lesion was not acute due to the marked intralesional fibrosis and vascular proliferation. Similar brain lesions were described in cerebral infarcts of sheep that were induced 43 days previously by experimental arterial ligation [26]. In human ischemic brain infarcts, migration of microglia/macrophages occurs 5–6 days after the onset of necrosis and capillary proliferations are noted after 5–10 days [25]. The presence of abundant fibrotic scar tissue was likely attributed to a breach in the meninges that allowed fibroblast invasion into the necrotic tissue [32]. In addition, the survival of pericytes within the infarcted area may have contributed to the fibrosis as well [27]. Similar as observed in the present case, cholesterol crystal deposition may also occur in necrotic areas of the human brain [33]. The cholesterol is mainly released from injured myelin sheaths and evokes a chronic inflammatory response that was also detected in the alpaca brain and further augments stroke associated brain injury [33].

The incidentally found focal brain lesion in this alpaca strongly suggests for a stroke of an either ischemic or hemorrhagic etiology, while the observed neuropathological hallmarks on the cellular level with reference to the neurovascular unit concept more emphasize the ischemic nature of the lesion. These results support further translational research, as they may help to explore stroke-caused cellular reactions beyond the rodents’ brain in more detail.

## Abbreviations

AQP4: aquaporin 4
C2 and C3: 2nd and 3rd stomach compartment
GFAP: glial fibrillary acidic protein
Iba: ionized calcium binding adapter protein 1
NeuN: neuronal nuclear antigen
NF-L: neurofilament light chain
STL: *Solanum tuberosum* lectin
TBS: Tris-buffered saline
